# 25OH vitamin D3 in biliary atresia: a simple and under-utilised diagnostic method

**DOI:** 10.3389/fped.2025.1658405

**Published:** 2025-09-19

**Authors:** Fangjuan Song, Wanfu Li, Zhida Chen, Ayiguzali Maimaijiang, Yaqi Wang

**Affiliations:** Department of Pediatric General Surgery, The First Affiliated Hospital of Xinjiang Medical University, Urumqi, China

**Keywords:** biliary atresia, children, 25(OH)D3, under-utilised, diagnostic

## Abstract

**Background:**

Biliary atresia is a form of pediatric cholangiopathy that affects the bile ducts of the liver. If left undiagnosed and untreated, it often leads to liver failure and death within 1–2 years of age. The treatment approach for this condition is recognized as the Kasai surgical procedure,which has been shown to achieve optimal outcomes when performed within 60 days of age. Furthermore, the timing of surgery has been identified as a crucial factor in determining patient prognosis. The current clinical practice involves the use of ultrasound as a primary diagnostic tool. Serum γ-glutamyltranspeptidase and other biochemical indicators are utilized in the diagnosis of biliary atresia, yet challenges persist regarding the occurrence of false-positive results. Consequently, there is an urgent clinical necessity to investigate non-invasive indicators for the early diagnosis of BA in children, with the objective of enhancing the accuracy of early diagnoses. In clinical practice, we frequently encounter cases of severe vitamin D deficiency in children diagnosed with BA. In recent years, the association between vitamin D and liver fibrosis in chronic liver disease has been substantiated. The objective of this study was to ascertain the clinical value and significance of 25(OH)D3 in the early diagnosis of BA.

**Objective:**

The best early diagnosis of BA is currently clinically inconclusive, and no test can confirm the diagnosis before surgery. To explore the clinical value of 25(OH)D3 in the diagnosis of biliary atresia (BA).

**Methods:**

The observation group comprised paediatric patients with biliary atresia admitted to the First Affiliated Hospital of Xinjiang Medical University between January 2024 and March 2025. The control group consisted of paediatric patients with cholestatic diseases caused by conditions other than biliary atresia during the same period.

**Results:**

There were significant differences in ultrasound results, serum γ-glutamyl transpeptidase level and 25(OH)D3 level between the BA group and the non-BA group (*P* < 0.05). The level of γ-glutamyl transpeptidase in the BA group was significantly higher than that in the non-BA group (*P* < 0.05), the level of direct bilirubin in the BA group was significantly higher than that in the non-BA group (*P* < 0.05), and the level of 25(OH)D3 in the BA group was significantly lower than that in the non-BA group (*P* < 0.05). A 25(OH)D3 cut-off level of 20.59 or lower combined with ultrasound results, or a 25(OH)D3 cut-off level of 20.59 or lower, ultrasound results, and serum γ-glutamyl transpeptidase cut-off levels of 283.89 or higher were combined to increase the sensitivity by 2.5%, specificity by 1.96%, PPV by 2.5%, NPV by 1.96%, and accuracy by 2.20% compared with 25(OH)D3 alone.

**Conclusions:**

The level of 25(OH)D3 can be used as a new force for diagnosing BA to provide an important detection basis for whether children need early surgical treatment, and is a good indicator for the diagnosis and differential diagnosis of BA.Moreover, the combined ultrasound results or 25(OH)D3 level, serum *γ*-glutamyl transpeptidase, and ultrasound results have higher diagnostic efficiency, and it is recommended to combine them for diagnosis.

## 1 Introduction

BA is a form of pediatric cholangiopathy that affects the bile ducts of the liver (1). If left undiagnosed and untreated, it often leads to liver failure and death within 1–2 years of age (2). Research has demonstrated that the prevalence of BA in Asia is significant, ranging from 100 to 500 cases per 100,000 live births (3, 4). The optimal treatment approach for this condition is recognized as the Kasai surgical procedure, which has been shown to achieve optimal outcomes when performed within 60 days of age. Furthermore, the timing of surgery has been identified as a crucial factor in determining patient prognosis. The current clinical practice in Asia involves the use of ultrasound as a primary diagnostic tool. Serum *γ*-glutamyl transpeptidase and other biochemical indicators are utilized in the diagnosis of BA, yet challenges persist regarding the occurrence of false-positive results. Consequently, there is an urgent clinical necessity to investigate non-invasive indicators for the early diagnosis of BA in children, with the objective of enhancing the accuracy of early diagnoses. In clinical practice, we frequently encounter cases of severe vitamin D deficiency in children diagnosed with BA. In recent years, the association between vitamin D and liver fibrosis in chronic liver disease has been substantiated. The objective of this study was to ascertain the clinical value and significance of 25(OH)D3 in the early diagnosis of BA.

## 2 Information and methods

### 2.1 General information

A total of 40 cases of children diagnosed with BA were selected for inclusion in the BA group. These cases were treated at the First Affiliated Hospital of Xinjiang Medical University from January 2024 to March 2025. The group comprised 18 male patients and 22 female patients. A further 51 children diagnosed with non-BA cholestatic diseases during the same period were selected for inclusion in the non-BA group as a control, with 24 males and 27 females. The study was conducted in accordance with the ethical standards for human experimentation, having been approved by the Ethics Committee of the First Affiliated Hospital of Xinjiang Medical University. Informed consent was obtained from the families of the subjects.

### 2.2 Inclusion and exclusion criteria

(1) Inclusion criteria: (1) The child did not undergo Kasai or liver transplantation; (2) The child is <100 days old; (3) Experimental group: biliary atresia, control group: common bile duct cyst, Alagille syndrome;

(2) Exclusion criteria: (1) Incomplete clinical data; (2) Cases with no clear diagnosis; (3) Children with metabolic diseases such as diabetes, hypothyroidism, hyperthyroidism, liver and kidney function, heart function diseases, and malignant tumors; (4) Underlying factors such as weak vitamin D absorption or vitamin D deficiency, such as premature infants, children with physiological jaundice, and progressive familial cholestasis diseases.

### 2.3 Research methods

General and clinical data collection included children's age, gender, weight, ultrasound results, preoperative serum γ-glutamyl transpeptidase level, and preoperative serum 25(OH)D3 level.

#### 2.3.1 Ultrasonography

Instrument: SuperSonic Aixplorer US ultrasound diagnostic instrument, SL10-2 probe, frequency 2–10 MHz.

Pre-examination preparation:Children: (1) 1 day before the child's Kasai or liver transplantation or on the second day of admission for children receiving conservative treatment; (2) fasting for 6 h; elastography examiner: a physician with more than 5 years of experience in diagnosing children's abdominal ultrasound; Preparation: when the child is unable to cooperate with the examination, oral chloral hydrate 1% 1 ml/kg or an enema is given for sedation.

Positive ultrasound examination criteria in this study: (1) gallbladder rigidity; (2) small gallbladder (<15 mm in length); (3) cysts in the porta hepatis; (4) TC hypoechoicity in the porta hepatis of ≥2 mm in thickness, i.e., the TC sign; (5) reduced CrGB (<50%); (6) hepatic arterial widening (≥2 mm in diameter); (7) elevated Young's modulus of the liver; (8) increased hepatic arteries (≥2 mm in diameter); (9) Increased hepatic Young's modulus (≥7.6 kPa). The presence of more than 2–3 positive signs is diagnostic of BA (5). [Gallbladder contraction rate (CrGB), CrGB = (Fasting gallbladder maximal cross-sectional area-Postprandial gallbladder maximal cross-sectional area)/Fasting gallbladder maximal cross-sectional area × 100%].

#### 2.3.2 Measurement of serum gamma-glutamyl transpeptidase level

In both groups, 3 ml of fasting venous blood was collected in the early morning, following centrifugation at a rate of 3000 r/min for 10 min. The upper layer of clear fluid was then extracted and placed in centrifugal tubes. This process was conducted prior to the administration of medication to the children within 24 h of their admission to the hospital. The serum γ-glutamyl transpeptidase level was measured by the rate method using an AU5800 automatic biochemical analyzer with accompanying reagents provided by Beckman Coulter Ltd. in the United States. In order to circumvent the occurrence of bias pertaining to disease progression, the temporal span between the collection of blood samples and the elastography examination was reduced to a duration of less than three days. This approach was adopted to ensure adherence to the stipulated consultation schedule (6).

#### 2.3.3 Measurement of serum 25(OH)D3 levels in children

##### 2.3.3.1 Detection method

(1) Detection target: 25(OH)D3; (2) Detection method:electrochemiluminescence; (3) Selection basis: high sensitivity, high specificity, convenient, fast, suitable for large sample analysis.

##### 2.3.3.2 Experimental principle and signal detection

(1) immunoreaction; (2) electrochemical excitation; (3) luminescence signal; (4) signal detection: photomultiplier to measure the luminescence intensity, and then automatically calculate the concentration through the cobas e 601 detector.

##### 2.3.3.3 Experimental steps

###### 2.3.3.3.1 Instruments and kits

(1) Instrument cobas e 601, SuperSonic Imagine, SA (Aix-en-Provence); (2) Kit Elecsys Vitamin D total III; (3) Key reagents Ruthenium-labeled antibody 25(OH)D3 antibody, biotinylated anti-25(OH)D3 antibody, streptavidin-coated magnetic microbeads, standards and quality control materials.

###### 2.3.3.3.2 Sample standards

(1) Samples are serum collected from standard sampling tubes; (2) Samples can be stored stably at 20–25 ℃ for 6 h, 2–8 ℃ for 3 days, and −24 ℃ for 20 weeks (can be frozen only once); (3) Samples, calibrators and QCs are equilibrated to about 20 ℃ before the test; (4) Samples, calibrators and QCs are required to perform the assay within one hour; (5) Samples with precipitation are centrifuged and tested. centrifuged for detection.

###### 2.3.3.3.3 Test principle

(1) Sample addition; (2) Magnetic separation; (3) Electrochemical excitation; (4) Signal reading.

###### 2.3.3.3.4 Test method

(1) Determination: Use the analyzer to read the bar code information. If it cannot be read, enter the 15-digit sequence on the bar code label.
(2) Calibration: Specific calibration information corresponding to the barcode batch of reagents; the calibration curve is applicable to the analyzer of the relevant kit. (Re-calibration is required: the analyzer uses the same reagent kit for more than seven days and the same batch number reagent kit for more than 10 weeks.)
(3) Quality control: Quality control of each concentration should be tested at least once every 24 h, and quality control should be repeated after replacing the reagent kits or calibrating. The samples should be repeated if necessary.
(4) Calculation: The analyzer can directly calculate the concentration of the test sample.

The grouping method involved the classification of all subjects into two distinct groups: BA and non-BA. The categorization of the BA group was determined by the etiology of the disease. The diagnosis of the BA group was confirmed through the utilization of cholangiography during caesarean section or laparoscopic exploration. In cases involving Kasai surgery or liver transplantation, the diagnosis was reconfirmed through the analysis of postoperative liver pathology. In the non-BA group, the etiology of the disease was elucidated by the aforementioned examinations, or alternatively, the etiology of the disease remained unclarified; yet, the condition underwent a progressive amelioration following conservative treatment, with the jaundice gradually subsiding during the subsequent follow-up.

### 2.4 Statistical analysis

The statistical analysis was conducted using SPSS 27.0. The measures that satisfied the assumptions of normality were expressed as the mean ± standard deviation, and comparisons between the two groups were made using the independent samples t-test. The measures that did not satisfy the assumptions of normality were expressed as the median (interquartile spacing) [M (P25, P75)], and comparisons between the two groups were analyzed using the Mann–Whitney U test. The count data were expressed as frequencies and component ratios, and intergroup comparisons of categorical variables were analyzed using the chi-square test. A statistically significant difference was indicated by *P* < 0.05.

## 3 Results

### 3.1 General comparison

There was no statistically significant difference between the two groups with regard to age and sex (*P* ≥ 0.05). (See Table 1).

**Table 1:** Comparison of general conditions of children in two groups.

Item	BA group (*n* = 40)	Non-BA group (*n* = 51)	Statistical value	*P*
Day age (d)	44.05 ± 5.01	42.29 ± 5.42	1.577	0.118
Sex			0.038	0.845
Male	18 (45%)	24 (47.1%)		
Female	22 (55%)	27 (52.9%)		
Weight (kg)	5.29 ± 0.64	5.32 ± 0.72	−0.191	0.114

### 3.2 Ultrasound results

A statistically significant difference in ultrasound findings was identified between the two groups (*P* < 0.05). The percentage of ultrasound findings diagnosing biliary atresia in the BA group was 92.5%, while the percentage diagnosing non-biliary atresia was 7.5%. In contrast, the percentage of ultrasound findings diagnosing biliary atresia in the non-BA group was 3.9%, and the percentage diagnosing non-biliary atresia was 96.1% (see Table 2). The sensitivity of its diagnosis of BA was 92.5%, the specificity was 94.12%, the positive predictive value (PPV) was 92.50%, the negative predictive value (NPV) was 94.12%, and the accuracy was 93.41% (see Table 3).

**Table 2:** Comparison of ultrasound results, serum γ-glutamyl transpeptidase, direct bilirubin and 25(OH)D3 between the two groups of children.

Individual indicators	BA group (*n* = 40)	Non-BA group (*n* = 51)	Statistical value	*P*
Ultrasound results			68.274	<0.05
BA	37 (92.5%)	3 (5.88%)		
Non-BA	3 (7.5%)	48 (94.12%)		
GGT (U/L)	807.45 (515.77, 973.43)	160.91 (64.91, 285.53)	−5.877	<0.05
DB (umol/L)	93.03 (88.57, 102.30)	0.3 (0.3, 16.43)	−7.138	<0.05
25(OH)D3 (nmol/L)	8.05 (7.71,12.15)	47.67 (41.52,52.43)	−8.116	<0.05

Serum γ-glutamyl transpeptidase, GGT; direct bilirubin, DB.

**Table 3:** Evaluation of the efficacy of ultrasound findings, serum γ-glutamyl transpeptidase, direct bilirubin, and 25(OH)D3 in the diagnosis of biliary atresia.

Individual indicators	Cut-off value	Sensitivity (%)	Specificity (%)	PPV (%)	NPV (%)	Accuracy (%)
Ultrasound results	–	92.5	94.12	92.50	94.12	93.41
GGT	283.89	90	78.43	76.60	86.27	83.52
DB	87.80	77.5	96.08	93.94	84.48	87.91
25(OH)D3	20.59	95	96.08	95.00	96.08	95.60

Serum γ-glutamyl transpeptidase, GGT; direct bilirubin, DB; PPV, positive predictive value; NPV, negative predictive value.

### 3.3 Serum γ-glutamyl transpeptidase level

A statistically significant difference in serum γ-glutamyl transpeptidase level was identified between the two groups (*P* < 0.05), with the serum level in the BA group exhibiting a marked increase compared to the non-BA group. The median serum γ-glutamyl transpeptidase level in the BA group was 807.45 (U/L), and the median serum γ-glutamyl transpeptidase level in the non-BA group was 160.91 (U/L). As illustrated in Table 2, a serum gamma-glutamyl transpeptidase level cutoff value of 283.89 or higher exhibited a diagnostic sensitivity of 90%, specificity of 78.43%, positive predictive value (PPV) of 76.60%, negative predictive value (NPV) of 86.27%, and accuracy of 83.52% for diagnosing BA (see Table 3).

### 3.4 Direct bilirubin level

A statistically significant difference in direct bilirubin levels was identified between the two groups (*P* < 0.05). Direct bilirubin levels in the BA group were found to be significantly higher than those in the non-BA group. The median direct bilirubin level in the BA group was 93.03 (μmol/L), while the median direct bilirubin level in the non-BA group was 0.30 (μmol/L) (see Table 2). A direct bilirubin level cutoff value of 87.80 or higher exhibited a diagnostic sensitivity of 77.5%, specificity of 96.08%, positive predictive value (PPV) of 93.94%, negative predictive value (NPV) of 85%, and accuracy of 87.91% for diagnosing BA (see Table 3).

### 3.5 Levels of 25(OH)D3

The variation in 25(OH)D3 levels between the two groups was found to be statistically significant (*P* < 0.05), with the 25(OH)D3 levels in the BA group being significantly lower than those in the non-BA group. The median 25(OH)D3 level was 8.05 (nmol/L) in the BA group and 47.67 (nmol/L) in the non-BA group (see Table 2). With a cutoff value of 20.59 or lower, the 25(OH)D3 level significantly differentiated between BA and non-BA cholestatic disorders, with a sensitivity of 95%, specificity of 96.08%, and positive predictive value (PPV) of 95.00%. The negative predictive value (NPV) was 96.08%, and the accuracy was 95.60%. Levels of 25(OH)D3 were found to differentiate between BA and non-BA cholestatic disorders. The diagnostic efficacy of 25(OH)D3 levels in distinguishing BA from non-BA cholestatic diseases was significantly enhanced in comparison to ultrasound results, serum γ-glutamyl transpeptidase levels, and direct bilirubin levels with regard to sensitivity, specificity, positive predictive value, negative predictive value, and accuracy (see Table 3).

It can be observed that in instances where the cutoff level of 25(OH)D3 of 20.59 or lower is combined with the ultrasound result, or the cutoff level of serum γ-glutamyl transpeptidase of 283, the resultant data can be analyzed. When the cutoff value is set at 89 or higher, the distinction between BA and non-BA cholestatic diseases is substantially enhanced, exhibiting a sensitivity of 97.5%, specificity of 98.04%, positive predictive value (PPV) of 97.50%, negative predictive value (NPV) of 98.04%, and overall accuracy of 97.80%. The sensitivities exhibited a 2.5% increase, the specificities a 1.96% increase, the positive predictive values (PPVs) a 2.5% increase, the negative predictive values (NPVs) a 1.96% increase, and the accuracies a 2.20% increase compared to the mean 25(OH)D3 levels alone for the diagnosis of BA. Furthermore, the combination of a 25(OH)D3 cutoff level of 20.59 or lower with a serum *γ*-glutamyl transpeptidase cutoff level of 283.89 or higher resulted in a diagnosis of BA with a sensitivity of 95%, a specificity of 96.08%, a positive predictive value of 97.50%, a negative predictive value of 96.15%, and an accuracy of 90.00%. The present study demonstrated that the proportion of the population with a positive test result for 25(OH)D3 was 2.5% higher than that for the combination of 25(OH)D3 and PPV. The negative likelihood ratio was 1.1% higher, and the accuracy was 5.60% lower (see Table 4).

**Table 4:** Evaluation of the efficacy of 25(OH)D3 combined with each index in the diagnosis of biliary atresia.

Combined indicators	Sensitivity (%)	Specificity (%)	PPV (%)	NPV (%)	Accuracy (%)
25(OH)D3 + ultrasound results	97.5	98.04	97.50	98.04	97.80
25(OH)D3 + ultrasound results + GGT	97.5	98.04	97.50	98.04	97.80
25(OH)D3 + ultrasound results + DB	95	96.08	97.50	96.15	90.00

Serum γ-glutamyl transpeptidase, GGT; direct bilirubin, DB; PPV, positive predictive value; NPV, negative predictive value.

## 4 Discussion

BA has been identified as the primary cause of neonatal jaundice (7), and Kasai surgery is regarded as the preferred treatment. The timing of surgery is directly associated with the patient's prognosis. However, early diagnosis remains challenging. Currently, the gold standard for diagnosing BA is intraoperative cholangiography, a procedure that carries inherent risks, is traumatic, and is costly. Consequently, it is not considered an appropriate method for the early diagnosis of BA. The analysis of blood biochemistry is a simple and safe procedure for children, enabling continuous and early monitoring. Consequently, the identification of straightforward and effective blood biochemistry indicators is a pivotal research area for the early diagnosis of BA (8). Nevertheless, the optimal method for the early diagnosis of BA remains to be determined, as no single test can currently provide unequivocal confirmation of the diagnosis prior to surgery.

The majority of previous studies have focused on the relationship between vitamin D and liver fibrosis, or with other liver function indicators. However, no scholars have utilized vitamin D to diagnose BA, and the present study is the first to utilize 25(OH)D3 levels to diagnose BA.

The primary source of vitamin D is the diet, and the vitamin D present in food is first converted to 25(OH)D in the liver by the processes of hydroxylation of CYP2R1 and CYP27A1. Children with BA have impaired vitamin D activation due to CYP2R1 deficiency. Vitamin D inactivation can promote the proliferation and activation of hematopoietic stem cells and participate in the development of BA liver fibrosis (9). As demonstrated in studies (10–13), vitamin D has been shown to reduce the expression of collagen and key pro-fibrotic factors in hepatic stellate cells (HSC), LX-2 cells, and mesenchymal pluripotent cells (MMCs). In addition, it has been observed to offer a certain degree of protection against hepatic fibrosis. Supplementation of vitamin D has been found to be a viable treatment for hepatic fibrosis, with improvements in liver function being reported in studies (14, 15). In the context of severe liver disease in children, a vicious cycle of exacerbation of hepatic fibrosis and impaired absorption and activation of vitamin D has been observed. Vitamin D has been shown to play a role in the progression of cirrhosis (16). Zhuang et al. (10) demonstrated that serum 25-(OH)D levels were diminished in children with BA, and that 25(OH)D levels exhibited a negative correlation with the severity of hepatic fibrosis. The 25(OH)D levels of 161 children with BA were documented, and staging of liver fibrosis was conducted on their pathological specimens. Peng et al (17) demonstrated a negative correlation between serum vitamin D levels and the severity of hepatic fibrosis in BA patients following the Kasai operation. Biswas et al (18) also demonstrated a statistically significant negative correlation between 25(OH)D and serum γ-glutamyltranspeptidase.

In this study, the 25(OH)D3 level in the BA group was found to be significantly lower than that in the non-BA group, with a cut-off value of 20.59 or lower. Furthermore, the study demonstrated the ability of the BA level to significantly differentiate between BA and non-BA cholestatic diseases, with a sensitivity of 95%, a specificity of 96.08%, a positive predictive value (PPV) of 95.00%, a negative predictive value (NPV) of 96.08%, and an accuracy of 95.60%. Therefore, it is of significant clinical importance for the distinction of BA from non-BA cholestatic diseases. The sensitivity, specificity, positive predictive value, negative predictive value, and accuracy of different diagnostic modalities for BA varied.The sensitivity of ultrasound for BA diagnosis was 92.5%, the specificity was 94.12%, the positive predictive value was 92.50%, the negative predictive value was 94.12%, and the accuracy was 93.41%. The serum *γ*-glutamyl transpeptidase cut-off value was 305 or higher, and the direct bilirubin level cut-off value was 87.80 or higher. It is worth mentioning that the combination of 25(OH)D3 cut-off value level with ultrasound results, or 25(OH)D3 cut-off value, ultrasound results, and serum γ-glutamyl transpeptidase cut-off level of 283.89 or higher increased the diagnostic BA sensitivity by 2.5%, the specificity increased by 1.96%, the PPV increased by 2.5%, the NPV increased by 1.96%, and the accuracy increased by 2.20% compared with the 25(OH)D3 level alone.

It is noteworthy that nearly 70% of the ultrasound examinations in this study were performed by the same physician who has been practicing ultrasound diagnosis for 20 years. This can greatly reduce the errors in the experimental results caused by the differences in the experience and ability of ultrasound physicians. The present study was limited to a single-center investigation, a factor that may have introduced bias. A multicenter study could be conducted to verify the results of this study. In recent years (19, 20), the diagnostic efficacy of serum MMP-7 level in the diagnosis of BA has attracted much attention, with a sensitivity of >90% and a specificity of >85%.In conclusion, the level of 25(OH)D3 can be used as a new tool in the diagnosis of BA. This provides a solid foundation for determining whether children require early surgical intervention. It is also a reliable indicator for the diagnosis and differential diagnosis of BA. Furthermore, the combination of ultrasound results or 25(OH)D3 level, serum γ-glutamyltranspeptidase, and ultrasound results is more effective. This combination is of higher value for the clinical diagnosis of BA.

## Data Availability

The raw data supporting the conclusions of this article will be made available by the authors, without undue reservation.
